# Repeated low-level red-light therapy for improving asthenopic symptoms and accommodation in presbyopia

**DOI:** 10.1080/07853890.2026.2663105

**Published:** 2026-04-28

**Authors:** Fan Song, Ziwei Zhao, Jian Zhang, Jijing Li, Yue Wu, Yanjun Liu, Pengju Li, Yueye Wang, Mengying Lai, Mingguang He, Yanxian Chen

**Affiliations:** a School of Optometry, The Hong Kong Polytechnic University, Kowloon, Hong Kong; b Research Centre for SHARP Vision (RCSV), The Hong Kong Polytechnic University, Kowloon, Hong Kong; c State Key Laboratory of Ophthalmology, Zhongshan Ophthalmic Center, Sun Yat-sen University, Guangzhou, China; d The Center for Eye and Vision Research (CEVR), Hong Kong

**Keywords:** Presbyopia, repeated low-level red light, accommodation amplitude, asthenopia, randomized controlled trial

## Abstract

**Background:**

To assess the short-term effectiveness of repeated low-level red light (RLRL) therapy in relieving asthenopia and enhancing accommodation in presbyopia.

**Methods:**

This randomized, parallel-group, double-masked clinical trial enrolled adults with presbyopia and self-reported asthenopia. Participants were allocated using computer-generated randomization and randomly assigned at a 1:1 ratio to RLRL or sham groups. Blinding included participants, examiners, assessors, and statisticians. The primary outcome was the change from baseline in the Computer Vision Syndrome Questionnaire (CVS-Q) score at day 31. Secondary outcomes were the change in accommodative amplitude (AA), Near Activity Visual Questionnaire (NAVQ) score, habitual near visual acuity, near-addition power, accommodative facility, positive and negative relative accommodation, binocular cross-cylinder response, and accommodative convergence-to-accommodation ratio. Continuous outcomes were analyzed using linear mixed-effects models.

**Results:**

Sixty-four of 66 randomized participants (aged 41–62 years) completed the 1-month trial. At day 31, RLRL showed greater improvement than sham in CVS-Q score (adjusted mean difference, -1.75 points; 95% CI, -3.10 to -0.39), binocular AA (1.09 D; 95% CI, 0.37 to 1.82), and NAVQ score (-8.07 points; 95% CI, -14.17 to -1.97). The effect on AA was most pronounced in a subgroup of eyes with baseline amplitude >2.0 D (adjusted mean difference 1.33 D; 95% CI 0.32–2.34). Other measures did not differ between groups at each visit. No treatment-related adverse events were reported. Adherence was similar between groups (mean compliance: 98.2% vs 97.5%).

**Conclusions:**

Short-term treatment with RLRL significantly reduced asthenopic symptoms and improved accommodative amplitude in individuals with presbyopia.

Trial registration: NCT06745661 (registered December 8, 2024).

## KEY MESSAGES

- Repeated low-level red light therapy significantly reduced asthenopic symptoms in adults with presbyopia after 1 month of treatment.
- The therapy improved accommodative amplitude and vision-related quality of life compared with sham treatment.
- RLRL offers a promising, non-invasive approach for managing visual fatigue in presbyopia without observed adverse effects.

## Introduction

Presbyopia, the age-related loss of accommodation, affects over 1.8 billion people worldwide and is a growing global public health issue [1]. Beyond blurred near vision, the condition diminishes quality of life by causing significant visual fatigue, hampering daily tasks essential for independence and productivity [1–3]. Uncorrected presbyopia alone is estimated to cause US$25.4 billion in annual global productivity losses [4].

Current presbyopia care can be categorized into three main types. Optical solutions like progressive lenses can reduce visual quality with peripheral distortion, or compromising stereopsis [5]. Pharmacologic treatments, such as miotic drops, offer short-term near vision improvement but may cause poor vision in dim light, and a slight risk of retinal problems [6]. Surgical methods, can lessen spectacle dependence yet irreversibly induce higher-order aberrations or stereopsis loss, never restore dynamic accommodation, and carry inherent risks [7,8]. These limitations underscore the need for a non-invasive therapy that improves accommodation and symptoms without compromising visual quality.

Subfoveal choroidal thickness and vessel density decline steadily with age [9,10]. Sustained near work, a common trigger of asthenopia, causes prolonged ciliary-muscle contraction, and has been associated with further choroidal thinning and reduced choroidal blood flow (ChBF) [11]. Similarly, uncorrected presbyopia, characterized by hyperopic retinal defocus during near tasks, is linked to reductions in choroidal thickness and perfusion [12]. We hypothesize that reduced ocular microcirculation, together with the high metabolic demand of sustained accommodation, may contribute to accommodative strain, accommodative dysfunction, and asthenopic symptoms in presbyopic adults [13,14]. Repeated low-level red-light (RLRL) therapy is a non-invasive intervention that has been shown primarily in pediatric myopia trials to safely thicken the choroid and increase chorioretinal blood flow [15–17]. Although adult evidence remains limited, a randomized study in myopic adults reported that 4-week RLRL therapy shortened axial length and was accompanied by improvements in choroidal blood flow [18]. Beyond myopia, photobiomodulation (PBM) has also been evaluated in adults, and repeated PBM treatment has been reported to improve visual acuity, contrast sensitivity, and anatomical outcomes in subjects with dry age-related macular degeneration [19]. However, the efficacy in adults is mixed. It is also reported that PBM showed no benefit in patients with intermediate AMD, although it improved scotopic thresholds in the normal ageing group [20]. Taken together, these data support testing whether, by improving ocular microcirculation, RLRL could alleviate accommodative strain and reduce symptoms in presbyopia.

In this research, we conducted a double-masked, randomized controlled trial aiming to assess the short-term (1-month) efficacy and safety on asthenopic symptoms and accommodation function of repeated RLRL therapy in adults with presbyopia.

## Methods

### Study design and setting

This was a 1-month, randomized, parallel-arm, double-masked clinical trial. Participants were enrolled from February to March 2025, and all follow-up visits were completed in May 2025 at the Optometry Clinic of The Hong Kong Polytechnic University. The study adhered to the tenets of the Declaration of Helsinki and received ethical approval from the Institutional Review Board of The Hong Kong Polytechnic University in October 2024 (approval number: HSEARS20240916007). The trial was registered with ClinicalTrials.gov (identifier: NCT06745661) and followed the CONSORT reporting guideline. The trial protocol is provided in Supplement 1. Written informed consent was obtained from all participants prior to enrollment.

### Eligibility criteria

Eligible participants were adults aged >40 years with presbyopia, self-reported symptoms of asthenopia, and no history of light therapy within the past 6 months. Presbyopia was defined as near visual acuity that does not meet an individual’s needs despite optimal distance correction [21]. Exclusion criteria included the presence of ocular conditions that could cause eye pain or headache (e.g. strabismus, glaucoma, ocular trauma, conjunctivitis, keratitis, iridocyclitis), self-reported migraine or other relevant diseases; severe cataract; systemic conditions such as epilepsy, photosensitivity, or seizure disorders; illiteracy; and an afterimage duration longer than 6 min.

### Randomization and masking

A researcher independent of participant recruitment generated the random allocation sequence in a 1:1 ratio for the intervention (RLRL therapy) and control (sham device) groups using Stata software (version 18; StataCorp LLC). Simple randomization (no blocking) was used. Allocation was concealed with opaque, sequentially numbered envelopes sealed with tamper-evident tape. Each participant was assigned an envelope according to the order of enrollment. The envelope was opened by the independent researcher after baseline assessments and confirmation of eligibility to determine group assignment. The participants, optometrists, ophthalmologists, clinical examination technicians, and statisticians were masked to the treatment allocation. Outcome assessors, optical coherence tomography (OCT) graders, and statisticians were independent of the device manufacturer. The manufacturer had no access to the raw data or analytical code. No formal assessment of blinding credibility (allocation-guess) was performed. However, if a participant experienced a severe adverse event, unmasking would be performed by the investigators to assess causality, and the study intervention would be discontinued if the event was deemed related to the treatment.

### Intervention

Both groups used a portable desktop device (Eyerising International) equipped with a semiconductor laser diode that emitted low-level red light at a wavelength of 650 nm and approximately 1600 lux of illuminance through the pupil to the fundus [22]. The optical power entering a 4-mm pupil (the maximum pupil diameter observed after 10 s of bright-light exposure) is 0.29 mW [15]. Participants in the intervention group received RLRL therapy (0.29 mW under the 4-mm pupil assumption), whereas those in the control group used a sham device (10% of the active device’s power; ∼0.03 mW under the same assumption). During treatment, participants positioned their eyes against the eye mask and fixated on the built-in red fixation point. The eye mask has a wrap-around design to prevent interference from external light.

Each participant was provided with their assigned device to use at home. Following the baseline examination, all participants were instructed to use the device twice daily for 3 min per session, with a minimum interval of 4 h between sessions, 7 days a week for 1 month. Follow-up examinations were conducted at 15- and 31-days post treatment.

### Outcome measures

The primary outcome was the change in Computer Vision Syndrome Questionnaire (CVS-Q) score at the 31-day follow-up visit [23]. Secondary outcomes were changes, over the same time points, in accommodation amplitude (AA); binocular accommodative facility (BAF); Near Activity Visual Questionnaire (NAVQ) [24] score; habitual near visual acuity (HNVA); near-addition power (ADD); positive and negative relative accommodation (PRA and NRA), obtained with a phoropter; accommodative response on binocular cross-cylinder testing (BCC, ±0.50 D); and the gradient accommodative convergence-to-accommodation (AC/A) ratio. Safety endpoints were evaluated at baseline and both follow-up visits. Assessments included best-corrected distance visual acuity (BCDVA) at 4 m, best-corrected near visual acuity (BCNVA) at 40 cm, slit-lamp examination, OCT, and systematic collection of participant-reported adverse events.

All examiners, including the study technician, optometrist, and ophthalmologist, completed joint training on standardized measurement protocols before participant enrollment. Each participant was assessed at approximately the same time of day across visits by trained examiners. The CVS-Q and NAVQ scores were obtained using online questionnaires administered by a trained technician at each visit. Higher CVS-Q scores indicate more severe visual fatigue, whereas higher NAVQ scores indicate poorer near-vision function in presbyopia. AA was measured using the push-up method with a near target placed at 40 cm under full distance correction and appropriate near addition, if required. BAF was recorded as cycles per minute during a 30-second ±1.00 D flipper test. HNVA was measured at 40 cm with a near LogMAR chart, and BCVA was measured at 4 m with a standard LogMAR chart. ADD was determined as the minimum additional lens power required to achieve best near visual acuity under full distance correction. NRA and PRA were measured using a phoropter with initial near addition set according to age, under full distance correction. The AC/A ratio was determined by the gradient method. BCC was measured at 40 cm using *a* ± 0.50 D cross cylinder at near pupillary distance. Macular and optic nerve head assessments were performed using swept-source OCT (DREAM OCT^™^, Intalight). OCTA scans were acquired using an Angio 6 × 6 mm protocol (512 × 512, R4; 4 repeated B-scans per position). Image quality was assessed using the signal strength index (SSI), and scans with SSI < 8 were repeated. Structural changes were defined a priori as any new retinal or optic nerve head abnormality compared with baseline (e.g. foveal ellipsoid zone disruption, edema/hemorrhage, or optic disc swelling). For further analysis, actual relative accommodation and AA values were calculated by subtracting the near addition used during testing. Additional prespecified secondary outcomes are described in the Supplement 2 (Appendix S1).

### Intervention compliance monitoring

Participants in both groups were provided with unique accounts to use the device. The device automatically logged the date and time of each session and uploaded the records to a centralized system for monitoring. Two staff members reviewed adherence weekly and sent reminders to participants who missed scheduled sessions. Adherence was calculated as the percentage of completed sessions out of the prescribed schedule (twice daily for 31 days). A compliance threshold was defined as ≥80% of prescribed sessions (≥50/62 sessions).

### Adverse events

At every visit, any ocular or systemic symptoms and adverse events were recorded. Adverse events were defined as flash blindness, transient glare, prolonged after-image, and any decrease in vision, but any other symptom was also documented. Two senior ophthalmologists masked to treatment allocation independently reviewed the OCT scans of the macula and optic nerve to confirm that no new fundus lesions had developed. Discrepancies were resolved by consensus.

### Sample size calculation

The primary outcome was the change in CVS-Q score at the 31-day follow-up. We assumed a standardized mean difference of Cohen’s *d* = 0.80. Using a two-sided α = 0.05 and 80% power, a sample size of 26 participants per group (52 in total) was required according to G*Power 3.1. To account for an anticipated 15% attrition, we planned to enroll 31 participants per group (62 in total).

### Statistical analyses

Symptom improvement was defined as the change in CVS-Q and NAVQ scores from baseline to the 31-day visit; accommodation improvement was assessed as the change in AA, BAF, and the ancillary accommodative parameters over the same interval. NAVQ scores were transformed to a 0–100 Rasch scale, with higher values signifying poorer visual quality [25]. Normality of continuous variables was evaluated with the Kolmogorov-Smirnov test. Categorical variables are presented as n (%), and continuous variables as mean ± SD. Depending on distribution, continuous outcomes were analyzed using paired t tests or Wilcoxon signed-rank tests for within-group comparisons, and Welch’s t tests or Mann–Whitney U tests for between-group comparisons. Outcomes were analyzed using both intention-to-treat (including all randomized participants) and per-protocol (including participants who completed both follow-up visits) approaches. Missing follow-up outcomes for participants with no post-baseline data were imputed using baseline observation carried forward (BOCF) for the primary ITT analysis. This approach was used as a conservative assumption of no change for participants with no post-baseline measurements. To examine treatment effects while accounting for repeated measurements, we fitted general linear mixed-effects models (GLMM) for CVS-Q, NAVQ, AA, BAF, and other accommodation-function outcomes, adjusting for baseline age, sex, and the corresponding baseline value of each outcome. Visit was treated as a categorical variable, and fixed effects included treatment group, visit, and the treatment × visit interaction. A participant-level random intercept was included to account for within-participant correlation (i.e. correlation was modeled through the random-effects structure). This covariate adjustment was specified prior to the analyses to improve precision. No formal adjustment was made for multiple comparisons across secondary outcomes. Therefore, secondary outcome analyses were considered exploratory. In addition, an exploratory post hoc analysis was performed to assess whether treatment effects differed by baseline AA, using AA > 2.00 D as the threshold.

All statistical analyses were performed with Stata, version 18 (StataCorp), and statistical significance was defined as a two-sided *p* < 0.05.

## Results

**Figure 1.**
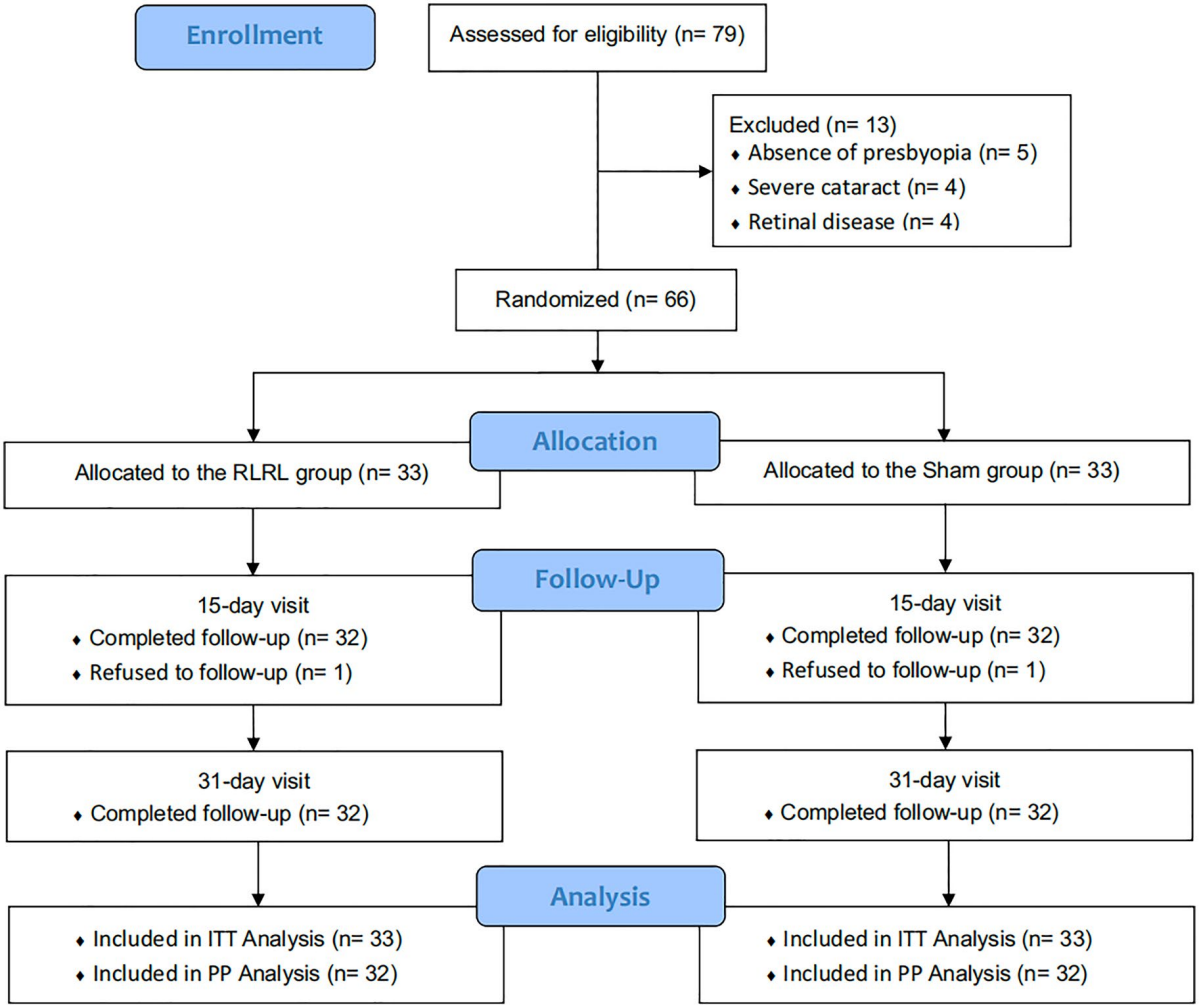
Flow diagram in this trial. RLRL = repeated low-level red-light therapy; ITT = intention to treat; PP = per protocol.

Of 66 eligible participants, 33 were randomized to RLRL therapy and 33 to sham treatment; 64 (97.0%) completed both follow-up visits (Figure 1). Baseline characteristics were comparable between groups (Table 1): mean age was 51.91 ± 5.74 years in the RLRL arm and 51.67 ± 5.02 years in the sham arm, and women represented 70% (*n* = 23) of each group. Other baseline variables, such as HNVA, ADD, SER, AA, AF, NRA, PRA, AC/A ratio, CVS-Q score, and NAVQ score, showed no significant between-group differences (all *p* > 0.05).

**Table 1.** Baseline characteristics of the included participants (intention-to-treat analysis).

Characteristic	RLRL Arm (*N* = 33)	Sham Arm (*N* = 33)
Age, years, mean (SD)	51.91 (5.74)	51.67 (5.02)
Female, No. (%)	23 (70)	23 (70)
Habitual near visual acuity, LogMAR, mean (SD)	0.13 (0.17)	0.12 (0.12)
Near ADD Required, No. (%)		
Mild presbyopia (≤ +1.25 D)	12 (36)	9 (27)
Moderate presbyopia(> 1.25 to +2.00D)	14 (42)	18 (55)
Advanced presbyopia(> +2.00D)	7 (21)	6 (18)
SER (OD), D, mean (SD)	−1.74 (2.60)	−2.31 (2.75)
AA (OU), D, mean (SD)	3.88 (2.60)	3.05 (1.96)
CVS-Q Score, mean (SD)	8.79 (3.50)	9.15 (4.62)
NAVQ Score (Rasch), mean (SD)	49.75 (17.70)	46.67 (17.99)

Abbreviations: AA = amplitude of accommodation; ADD = additional near-vision plus lens power; D = diopter; LogMAR = logarithm of the minimum angle of resolution; CVS-Q = Computer Vision Syndrome Questionnaire; NAVQ = Near Activity Visual Questionnaire; OD = right eye; OU = both eyes; RLRL = repeated low-level red-light therapy; SER = spherical equivalent refraction.

### Primary outcome

#### Changes in CVS-Q score

The reduction in the CVS-Q score was greater in the RLRL group than in the sham group (Table 2). From baseline to day 31, the unadjusted mean reduction in CVS-Q score was 2.58 points in the RLRL group vs. 0.73 points in the sham group (between-group difference in change, −1.85 points; 95% CI, −3.59 to −0.11; *p* = 0.038; Table 2). In the prespecified ITT linear mixed-effects model, the adjusted between-group difference in change at day 31 was −1.75 (95% CI, −3.10 to −0.39; *p* = 0.011), favoring RLRL (Table 3, Figure S1).

**Table 2.** Unadjusted mean changes in CVS-Q score in RLRL and sham groups.

Variable	RLRL	Sham	Mean difference (95% CI)	P Value
	Mean (SD)	Mean (SD)		
CVS-Q Score				
Change at 15 days				
ITT Analysis (*n* = 33)	−1.85 (3.99)	−0.45 (3.62)	−1.39 (−3.27, 0.48)	0.142
PP Analysis (*n* = 32)	−1.91 (4.04)	−0.47 (3.68)	−1.44 (−3.37, 0.49)	0.142
Change at 31 days				
ITT Analysis (*n* = 33)	−2.58 (3.45)	−0.73 (3.63)	−1.85(−3.59, −0.11)	0.038
PP Analysis (*n* = 32)	−2.66 (3.47)	−0.75 (3.69)	−1.91 (−3.70, −0.12)	0.037

Abbreviations: CVS-Q = Computer Vision Syndrome Questionnaire; ITT = intention-to-treat; PP = per-protocol.

**Table 3.** Cumulative adjusted mean changes in CVS-Q score from baseline to 15 and 31 days in RLRL group and sham group.

Outcome	Visit/Group	Cumulative adjusted mean change of outcomes (95% CI)		Mean difference (95% CI)	P Value
		RLRL Arm	Sham Arm		
CVS-Q	15 Days	−1.80 (−2.76, −0.84)	−0.50 (−1.46, 0.46)	−1.30 (−2.66, 0.05)	0.059
	31 Days	−2.53 (−3.48, −1.57)	−0.78 (−1.74, 0.17)	−1.75 (−3.10, −0.39)	0.011

Note: Values are from generalized linear mixed models (GLMMs) adjusted for baseline score, age, and sex. Mean differences represent between-group comparisons at each visit. CVS-Q = Computer Vision Syndrome Questionnaire; CI = confidence interval.

### Secondary outcomes

#### Changes in accommodative function

At 31-day follow-up, the RLRL group demonstrated significantly greater improvement in binocular AA compared to the sham group, with an adjusted mean change of 1.29 D (95% CI, 0.77 to 1.80) versus 0.19 D (95% CI, −0.32 to 0.70), yielding an adjusted mean between-group difference of 1.09 D (95% CI, 0.37 to 1.82; *p* = 0.003; Table 4). This treatment effect was consistent monocularly, with the right eye (OD) showing a 0.69 D greater improvement (95% CI, 0.10 to 1.29) and the left eye (OS) a 1.09 D improvement (95% CI, 0.50 to 1.69) in the RLRL group versus sham at 31 days.

**Table 4.** Cumulative adjusted mean changes in accommodation function from baseline to 15 days and 31 days between RLRL group and sham group.

Outcomes	Visit/Group	Cumulative adjusted mean change of outcomes (95% CI)		Mean difference (95% CI)	P Value
		RLRL Arm	Sham Arm		
AA (OD), D	15 Days	0.52 (0.10, 0.95)	0.02 (−0.40, 0.44)	0.50 (−0.10, 1.10)	0.100
	31 Days	0.84 (0.41, 1.26)	0.14 (−0.28, 0.57)	0.69 (0.10, 1.29)	0.023
AA (OS), D	15 Days	0.53 (0.11, 0.96)	−0.26 (−0.68, 0.16)	0.79 (0.20, 1.39)	0.009
	31 Days	1.00 (0.58, 1.42)	−0.09 (−0.51, 0.33)	1.09 (0.50, 1.69)	<0.001
AA (OU), D	15 Days	0.58 (0.07, 1.09)	−0.06 (−0.57, 0.46)	0.63 (−0.09, 1.36)	0.086
	31 Days	1.29 (0.77, 1.80)	0.19 (−0.32, 0.70)	1.09 (0.37, 1.82)	0.003
AF (OU), cpm/30s	15 Days	1.56 (0.84, 2.28)	0.56 (−0.16, 1.28)	1.00 (−0.02, 2.03)	0.054
	31 Days	1.97 (1.25, 2.69)	1.56 (0.84, 2.28)	0.41 (−0.61, 1.43)	0.434
NRA, D	15 Days	0.20 (0.00, 0.39)	0.12 (−0.07, 0.31)	0.08 (−0.19, 0.35)	0.572
	31 Days	0.38 (0.18, 0.57)	0.42 (0.23, 0.62)	−0.05 (−0.32, 0.22)	0.724
PRA, D	15 Days	−0.05 (−0.19, 0.10)	0.19 (0.05, 0.33)	−0.23 (−0.44, −0.03)	0.023
	31 Days	−0.01 (−0.15, 0.14)	−0.00 (−0.14, 0.14)	−0.00 (−0.21, 0.20)	0.966
BCC, D	15 Days	0.43 (0.28, 0.58)	0.59 (0.44, 0.75)	−0.17 (−0.38, 0.05)	0.126
	31 Days	0.59 (0.44, 0.74)	0.64 (0.49, 0.79)	−0.05 (−0.26, 0.17)	0.678
AC/A ratio	15 Days	−0.29 (−1.00, 0.42)	−0.63 (−1.33, 0.07)	0.34 (−0.66, 1.34)	0.504
	31 Days	−1.17 (−1.88, −0.45)	−0.79 (−1.49, −0.09)	−0.38 (−1.38, 0.62)	0.459

Note: Mean differences represent between-group comparisons at each visit. AA = accommodative amplitude; AF = accommodative facility; NRA = negative relative accommodation; PRA = positive relative accommodation; BCC = binocular cross-cylinder; AC/A = accommodative convergence to accommodation ratio; OD = right eye; OS = left eye; OU = both eyes/binocular; D = diopters; cpm/30s = cycles per minute measured over a 30-second interval; CI = confidence interval.

**Figure 2.**
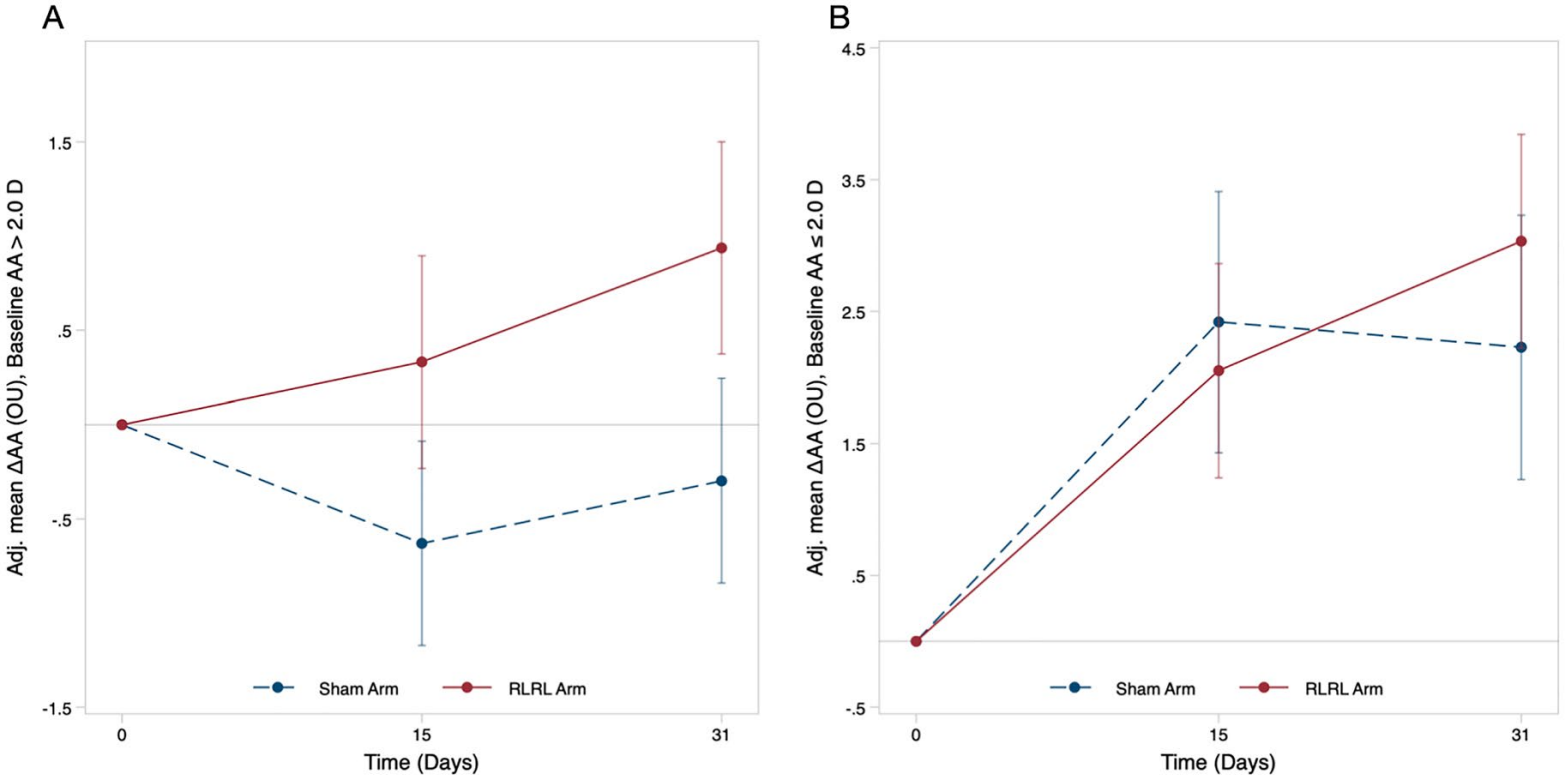
Adjusted mean change in accommodative amplitude (AA) by baseline AA level at follow-up visits. Error bars represent 95% confidence intervals (CIs). Adj. = adjusted; AA = accommodative amplitude; D = diopters; SER = spherical equivalent refraction; CI = confidence interval.

A subgroup analysis based on baseline AA revealed that the treatment effect appeared larger in participants with higher residual accommodation (Figure 2, Table S1). Among participants with a baseline AA >2.0 D, the adjusted mean difference in binocular AA change between the RLRL and sham groups at 31 days was 1.32 D (95% CI, 0.50 to 2.14; *p* = 0.002). In contrast, for participants with a baseline AA ≤ 2.0 D, the between-group difference was not statistically significant (0.80 D; 95% CI, −0.81 to 2.42; *p* = 0.329). The treatment-by-subgroup interaction at 31 days was significant for AA in the left eye (P for interaction = 0.011) and borderline in the right eye (P for interaction = 0.053).

Regarding other accommodative parameters (i.e. binocular AF, NRA, BCC, and the AC/A ratio), no significant differences in change were found between the RLRL and Sham groups at 31 days. In covariate-adjusted models, age and SER were not significantly associated with changes in AA, AF, or NAVQ, and inclusion of SER did not materially alter the estimated RLRL-Sham differences. Detailed results for all accommodative functions at 15 and 31 days are presented in Table 4, Table S2, Table S3, and Table S4.

#### Changes In near visual ability

**Figure 3.**
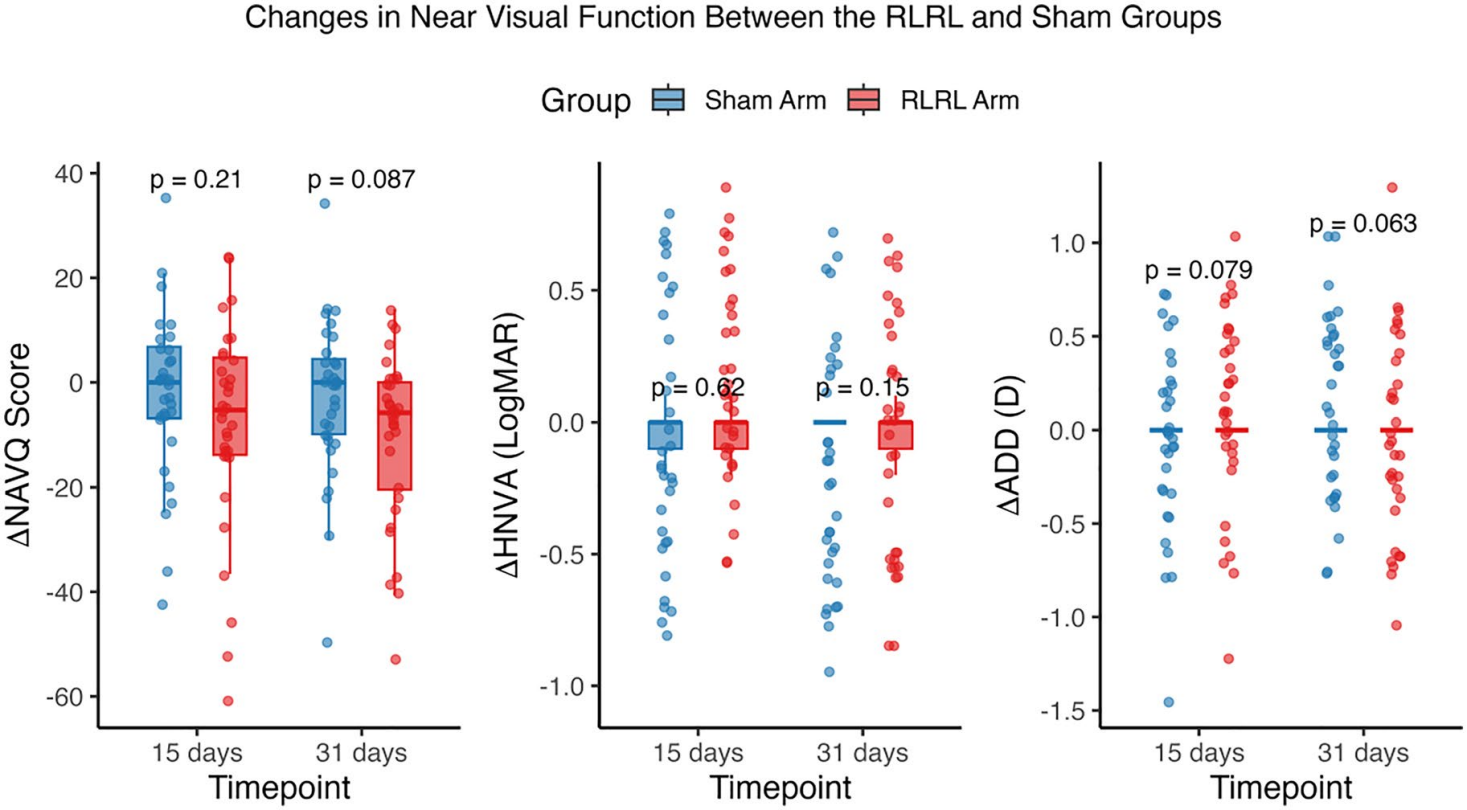
Unadjusted mean change of near visual function (NAVQ score, HNVA, and ADD) from baseline to day 15 and day 31 between RLRL and Sham groups. Negative values indicate improvement for NAVQ, HNVA, and ADD. Error bars represent 95% CIs. NAVQ = Near Activity Visual Questionnaire; HNVA, habitual near visual acuity; ADD = near-addition power; D = diopter; CI = confidence interval.

Participants receiving RLRL therapy showed significantly greater improvement in near visual ability compared with the sham group. The adjusted mean reduction in NAVQ score was −6.67 points (95% CI, −12.79 to −0.59; *p* = 0.032) at 15 days and −8.07 points (95% CI, −14.17 to −1.97; *p* = 0.009) at 31 days (Table S5). Unadjusted Wilcoxon rank-sum tests demonstrated the same direction of effect but did not reach statistical significance (*p* = 0.206 at 15 days; *p* = 0.086 at 31 days; Figure 3 and Table S6). No significant between-group differences were observed in other measures of near visual function, including HNVA and ADD (Figure 3, Table S6).

#### Compliance

Adherence was high and comparable between groups. The mean compliance rate was 98.2% (range, 88.7%–100.0%) in the RLRL group and 97.5% (range, 87.1%–100.0%) in the Sham group (*p* = 0.149). No device malfunctions or mid-trial reprogramming were recorded during the trial. Details are provided in Table S7.

#### Adverse events

No subjective adverse events were reported by participants in either the RLRL or control group throughout the trial. At the 1-month follow-up, all participants achieved a BCNVA of 0.0 LogMAR. Two participants in the RLRL arm (6.1%) and one in the control arm (3.0%) did not reach 0.0 LogMAR BCDVA at day 31; all three achieved 0.1 LogMAR. All cases of 0.1 logMAR BCDVA were unilateral (right eye), not accompanied by subjective visual symptoms, and were considered within normal measurement variability. Furthermore, serial OCT imaging revealed no structural signs of retinal or optic nerve abnormalities in any participant.

## Discussion

To our knowledge, this trial is the first to show that RLRL therapy alleviates asthenopic symptoms and enhances accommodative performance in adults with presbyopia. Over 31 days, the therapy produced a meaningful reduction in self-reported visual fatigue, with an adjusted mean reduction in the CVS-Q score of 2.53 points for the RLRL group versus 0.78 for sham. This subjective improvement was accompanied by a significant gain in functional near-vision ability, as measured by an 8.07-point between-group difference on the NAVQ.

Our finding of a 2.53-point reduction in the CVS-Q score is particularly noteworthy in an area where high-certainty evidence for any intervention remains scarce [26]. Test-retest repeatability of the CVS-Q has been reported to be good in an older population, and a minimum clinically important difference of 1.96 has been proposed [27]. For comparison, common advice like the 20-20-20 rule offers little benefit [28], and widely marketed products like blue-light filtering glasses are not supported by strong evidence [26]. Furthermore, alongside symptom relief, we observed an improvement in functional near vision: the mean NAVQ score decreased by 10.49 points within the RLRL arm at day 31, suggesting a clinically meaningful improvement [29]. While the functional improvement in NAVQ is more modest than that achieved with surgical interventions like presbyopia LASIK [25], it represents a significant, non-invasive gain. The benefit is biologically plausible, as low-level red light has been reported to lessen ocular-surface inflammation and dry-eye discomfort, which commonly contribute to digital eyestrain [30,31].

The objective data support the subjective gains. After one month, binocular AA was 1.08 D higher in the RLRL arm than with sham, echoing the accommodative improvement reported by Chen et al. [32] This gain may reflect an enhancement of the accommodative apparatus, in contrast to miotic agents like pilocarpine 1.25%, whose reported 0.69 D increase is largely attributed to an optical pinhole effect [33]. Our subgroup analysis further revealed that this benefit was primarily observed in eyes that still possessed accommodative reserve (baseline AA >2.0 D), suggesting that RLRL may augment the native accommodative mechanism but may be less effective in advanced lenticular sclerosis [34]. Clinically, extra one to two diopters of accommodation would meet the needs many normal near tasks [34], underscoring the value of early intervention. However, published data suggest that the 95% repeatability limits for push-up AA are approximately 1.4 D, indicating that an approximately 1-diopter change should be interpreted cautiously [35].

Several photobiomodulation mechanisms may explain these benefits. First, RLRL may enhance intraocular hemodynamics. Prior studies have reported that red light rapidly boosts retinal perfusion, increases choroidal blood flow, and induces nitric oxide release, potentially improving intraocular circulation and oxygen supply [16,36]. S, at a cellular level, RLRL optimizes energy metabolism. It has been shown to increase mitochondrial ATP production in retinal neurons [37] and, in animal models, to inhibit oxidative stress and retinal cell death [38]. Additionally, red light can induce nitric oxide release, leading to vasodilation and further improved blood flow [39]. Collectively, these findings suggest that RLRL may improve the physiological efficiency of the accommodative system. We hypothesize that, by increasing the oxygen and energy supply to the ciliary muscle, RLRL could reduce the strain required to meet accommodative demands, thereby mitigating asthenopia symptoms.

The current management of presbyopia lacks a universally accepted restorative treatment. Existing strategies are primarily compensatory and carry significant trade-offs. Optical solutions like progressive lenses, for instance, add external power but introduce peripheral distortion without addressing the underlying pathophysiology [40]. Pharmacologic agents such as pilocarpine 1.25% induce a transient pinhole effect rather than restoring true accommodation and are associated with side effects like headache and dim vision [33,41]. Finally, surgical interventions permanently alter ocular optics, risking reduced contrast sensitivity, night vision disturbances, or the need for explantation [42,43]. In this context, RLRL therapy is non-invasive, may influence ocular physiology, and produced a 1.29 D accommodative gain after one-month, without these significant disadvantages. Importantly, these benefits were achieved without compromising visual acuity or accelerating the need for stronger reading corrections.

No serious adverse events occurred in our study, consistent with prior trials of RLRL for myopia control [17,22]. A case of retinal damage following a 5-month RLRL treatment was reported, demonstrating a decrease in visual acuity and disruption of the foveal ellipsoid zone in the retina. Both the visual acuity and the retinal structure partially recovered after discontinuation of the treatment [44]. One retrospective study reported a parafoveal cone-density decrease after about a year of RLRL exposure, with relative reductions of 4.76% to 7.51% at 0.2–0.5 mm eccentricities compared to controls (*p* < 0.05) [45]. However, this reduction in cone density may be within functional compensatory and indicate little clinical significance, as visual acuity preserved despite ≥38% density loss [46]. Overall, severe adverse reactions to RLRL are rare, but further monitoring and research are warranted to fully understand its long-term effects.

Several limitations warrant consideration. First, the 1-month follow-up precludes conclusions about the long-term durability of symptom relief and accommodative gains. In addition, AA was assessed using the examiner-dependent push-up method, and objective accommodative measurements (e.g. dynamic retinoscopy, or defocus curves) were not collected, limiting physiologic interpretation of the observed changes. Although pediatric myopia trials with up to two-years follow-up have generally reported no serious adverse events, long-term safety evidence for RLRL in a presbyopic population is lacking. Second, our sample-size calculation assumed a relatively large effect without pilot study data, which may limit the robustness of our effect estimates. In addition, the modest sample size limits subgroup precision and the ability to detect uncommon adverse events. Third, the single-center design may restrict generalizability. Fourth, the allocation-guess assessment was not formally performed. Given the lower irradiance of the sham device, perceptible differences in brightness may have affected masking and subjective outcomes (CVS-Q and NAVQ). In addition, physiological fluctuations in pupil diameter and accommodative responses during exposure may have introduced uncontrolled variability in effective retinal irradiance. Fifth, the study did not include head-to-head comparisons with existing presbyopia treatments such as topical miotics, multifocal optics, or vision therapy. Multicentre trials with larger cohorts, dose-response optimization, and direct comparators are needed to establish the durability, optimal parameters, and relative efficacy of RLRL therapy. Finally, several prespecified objective secondary outcomes were not reported in this manuscript (see Supplement 2, Appendix S1), which limits a comprehensive assessment of mechanistic and ancillary effects.

## Conclusions

RLRL reduced asthenopic symptoms and improved accommodative function versus sham in adults with presbyopia. Eyes with greater residual accommodative reserve responded most strongly, suggesting particular value in early presbyopia. As a non-invasive intervention, RLRL offers a compelling alternative to passive optical aids and treatments constrained by side-effects. Longitudinal studies are needed to confirm the durability of these gains and to establish optimal long-term dosing protocols.

## Supplementary Material

Supplement 1_Trial Protocol.docx

Supplement 2_Supplementary Online Content.docx

## Data Availability

The individual de-identified participant data, statistical code and any other materials can be accessed upon request from the corresponding author.
