# Supplementary material for: Repeated low-level red-light therapy for improving asthenopic symptoms and accommodation in presbyopia
Source: Ann Med. 2026 Apr 28;58(1):2663105. doi: 10.1080/07853890.2026.2663105 (PMC13126939; doi:10.1080/07853890.2026.2663105)
Supplement: Supplement 1_Trial Protocol.docx [file IANN_A_2663105_SM8668.docx]

| **Supplement 1**  **TRIAL PROTOCOL** |
| --- |
| **Investigating the Impact of RLRL on Alleviating Visual Fatigue in Presbyopic Population** |
| Version: 1.0  Date:15/09/2024 |
| **Principal Investigator:**  **Prof Mingguang He** |
| **Statement of Compliance**  This study will be conducted in compliance with all stipulation of this protocol, the conditions of the ethics committee approval, the Note for Guidance on Good Clinical Practice (ISO 14155:2020; MDR 2017/745). |

Tables of Contents

[1.Introduction 3](#_Toc202368366)

[2. Study Objectives 4](#_Toc202368367)

[2.1 Hypothesis 4](#_Toc202368368)

[2.2 Study Aims 5](#_Toc202368369)

[2.3 Outcome Measures 5](#_Toc202368370)

[3. Study Population 6](#_Toc202368371)

[3.1 Recruitment Procedure 6](#_Toc202368372)

[3.2 Inclusion Criteria 6](#_Toc202368373)

[3.3 Exclusion Criteria 6](#_Toc202368374)

[3.4 Consent 6](#_Toc202368375)

[4. Study Design 7](#_Toc202368376)

[4.1 Study Type and Design 7](#_Toc202368377)

[4.2 Randomization and Masking 7](#_Toc202368378)

[4.3 Intervention Compliance Monitoring 8](#_Toc202368379)

[4.4 Schedule and Ethical Consideration 8](#_Toc202368380)

[4.5 Study Methodology 8](#_Toc202368381)

[5. Participants Safety and Withdrawal 11](#_Toc202368382)

[5.1 Risk Management and Safety 11](#_Toc202368383)

[5.2 Handling of Withdrawals 11](#_Toc202368384)

[5.3 Replacement 12](#_Toc202368385)

[5.4 Protocol Waivers or Deviations Justification 12](#_Toc202368386)

[6. Statistical Methods 12](#_Toc202368387)

[6.1 Sample Size Estimation & Justification 12](#_Toc202368388)

[6.2 Power Calculations 12](#_Toc202368389)

[6.3 Statistical Methods to Be Undertaken 12](#_Toc202368390)

[7. Storage of Blood and Tissue Samples 13](#_Toc202368391)

[8. Data Security & Handling 13](#_Toc202368392)

[8.1 Where Records Will Be Kept & How Long Will They Be Stored 13](#_Toc202368393)

[8.2 Confidentiality and Security 13](#_Toc202368394)

[8.3 Ancillary Data 13](#_Toc202368395)

[9. References 14](#_Toc202368396)

# **1.Introduction**

Visual fatigue, or asthenopia, occurs when visual demands exceed the capacity of the visual system, leading to various ocular discomfort and systemic symptoms. This condition can negatively affect personal activities, quality of life, and work productivity.^1,2^ The increasing prevalence of asthenopia underscores its growing significance as a public health issue.^3^ In a study of Chinese ophthalmologists, over 40% experienced visual fatigue, with presbyopia identified as a major risk factor.^4^ Moreover, in a study of presbyopic computer workers, all participants reported experiencing at least one symptom of visual fatigue.^5^ Given the high prevalence of asthenopia in presbyopic population, finding effective methods to alleviate these symptoms is crucial.

Previous studies have demonstrated that exposure to red light wavelengths may alleviate symptoms of visual fatigue, such as eye redness and dry eye^6^, and may also support retinal health through neuroprotective effects^7^. Additionally, studies have shown that improvements in ocular microcirculation have been significantly associated with the relief of asthenopia.^8,9^ Repeated low-level red-light therapy (RLRL) can significantly increase ocular blood flow. Several studies consistently reporting a 10-20% thickening of the posterior choroid following red light therapy.^10-13^ The effect of RLRL on increasing choroidal blood flow has also been validated in myopic adults. In our preliminary study, we conducted a crossover randomized controlled study involving 37 adult myopic patients who underwent one month of RLRL treatment (ClinicalTrials.gov identifier: NCT05747742). The results showed a significant increase in peripapillary choroidal thickness (pChT), macular choroidal thickness (mChT), and the density of the peripapillary deep capillary plexus (DCP) compared to baseline (Figure 1). These findings suggest that RLRL improves ocular blood flow, potentially alleviating visual fatigue. Moreover, photobiomodulation (PBM), which incorporates the use of red light, has been shown to enhance regional cerebral blood flow, as well as improving mood, memory, and cognitive function.^14,15^ Despite these potential benefits, no research to date has explored the use of red light therapy specifically for alleviating visual fatigue in individuals with presbyopia.

In this study, we aim to evaluate the effects of RLRL therapy on alleviating visual fatigue in individuals with presbyopia over a one-month period. Additionally, we will assess the potential benefits on accommodation function, near visual ability, cognition, and emotional effects of RLRL in this population.

Figure 1. Changes in Choroidal Thickness and Capillary Density During One Month of RLRL Therapy


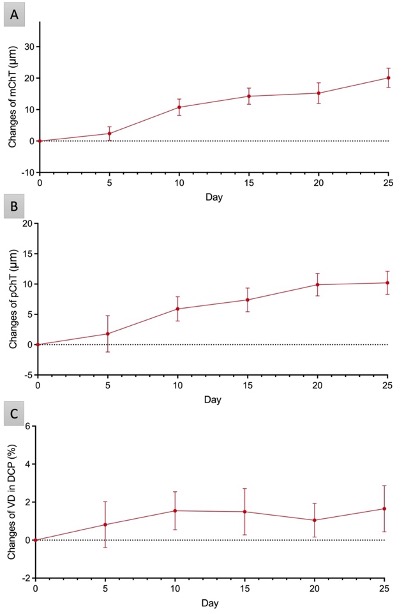


# **2. Study Objectives**

## 2.1 Hypothesis

RLRL therapy will effectively alleviate symptoms of asthenopia and well-being in individuals with presbyopia.

## 2.2 Study Aims

- To evaluate the effectiveness of RLRL therapy in reducing symptoms of asthenopia.
- To assess the impact of RLRL therapy on accommodation function, near visual ability, cognition, and emotional well-being in individuals with presbyopia.

## 2.3 Outcome Measures

### Primary Outcome

The primary outcome was the change in Computer Vision Syndrome Questionnaire (CVS-Q) score at the 31-day follow-up visit. ^16^ These scores will be derived from the Computer Vision Syndrome Questionnaire (CVS-Q), a widely recognized tool for assessing subjective symptoms of asthenopia.

### Secondary Outcomes

- Change in accommodation function, including accommodation amplitude (AA), facility of accommodation (AF), binocular cross-cylinder response (BCC), positive and negative relative accommodation (PRA, NRA), and accommodative convergence-to-accommodation (AC/A) ratio at 1 month compared to baseline.
- Change in near visual ability, including Near Activity Visual Questionnaire (NAVQ) score^17^, habitual near visual acuity (HNVA), near-addition power (ADD) at 1 month compared to baseline.
- Change in critical flicker fusion frequency (CFF) and other objective asthenopia indicators (e.g., eye movement, pupil parameters, and blink patterns) at 1 month compared to baseline.
- Change in cognition function, measured by EEG and the Montreal Cognitive Assessment Scale (MoCA)^18^ at 1 month compared to baseline.
- Change in self-reported emotional states, assessed by the Positive and Negative Affect Schedule (PANAS-X)^19^ at 1 month compared to baseline.

# **3. Study Population**

## 3.1 Recruitment Procedure

Interested individuals will be invited to the PolyU Optometry Clinic for eligibility screening. The research team will assess prospective participants through a series of evaluations, including visual acuity tests, slit lamp examinations, accommodation function examinations, fundus examinations, and a review of their medical history. Those who meet the eligibility criteria will proceed with the study procedures.

## 3.2 Inclusion Criteria

- Individuals aged over 40 years old.
- Self-reported symptoms of visual fatigue.
- Diagnosis of presbyopia, defined as near visual acuity that does not meet an individual’s needs despite optimal distance correction.
- Require near correction in daily life.
- No light therapy received in the past six months.
- Giving informed written consent.

## 3.3 Exclusion Criteria

- Presence of diseases that can cause eye pain or headaches, such as strabismus, glaucoma, ocular trauma, conjunctivitis, migraines, keratitis, iridocyclitis, or other self-reported diseases.
- Systemic diseases (e.g., epilepsy, photosensitivity, seizure) or illiteracy.
- Severe cataract.
- Afterimage time longer than 6 minutes.

## 3.4 Consent

In this study, the informed consent procedures and related documentation must first be approved by the ethics committee. This consent process will ensure eligible subjects are fully informed about the study's purpose, procedures, potential benefits, and risks. Researchers will thoroughly explain the nature of the study, with a focus on aspects related to visual fatigue in individuals with presbyopia.

Subjects will be encouraged to ask any questions they may have about the study, its methods, and its objectives, both before enrollment and throughout their participation. They will be clearly informed of their right to withdraw from the study at any point, without needing to provide a justification for their decision. Ensuring that the participants understand their involvement and rights within the study is a priority, to maintain ethical standards and participant comfort.

# **4. Study Design**

## 4.1 Study Type and Design

This prospective, randomized controlled study will enroll individuals aged 40 years or older who have asthenopia and presbyopia. The study aims to evaluate the effectiveness of RLRL therapy in reducing asthenopia and presbyopia symptoms, as well as its impact on cognitive function and emotional well-being.

Eligible participants will be randomly assigned in a 1:1 ratio to either the intervention group or the control group. Each participant in the intervention group will be provided an RLRL therapy device, which they are required to use twice daily for 3 minutes per session, with a minimum interval of 4 hours between sessions, 7 days a week for one month. Participants in the control group will receive a sham device, following the same usage schedule. The study will evaluate changes in asthenopia questionnaire scores, near visual ability, other objective asthenopia indicators, accommodation function, cognitive function, and emotional states at 2 weeks and 1 month compared to baseline.

## 4.2 Randomization and Masking

Eligible participants will be randomly assigned in a 1:1 ratio to either the intervention group or the control group, using a computer-generated randomization sequence. The intervention group will undergo RLRL therapy, while the control group will use a sham device, which operates at only 10% of the active device’s power. The light power through a 4-mm pupil is 0.29 mW for the RLRL device and 0.03 mW for the sham device. This therapy will be administered using a desktop semiconductor therapy device developed by Eyerising International Pty Ltd, based in Sydney, Australia.

Group assignments were concealed using opaque, sealed, and sequentially numbered envelopes. Each participant was assigned a number according to the order of enrollment, and the corresponding envelope was opened at the time of allocation to determine group assignment. Participants, optometrists, ophthalmologists, clinical examiners, and statisticians were all masked to the treatment allocation. However, if a participant experiences severe adverse reactions, the blinding may be lifted after the investigator determines that unblinding is necessary.

## 4.3 Intervention Compliance Monitoring

Each participant will receive an RLRL therapy device or a sham device and must log in with their unique account details before each treatment session. The device is connected to a centralized system via the internet, allowing for remote monitoring of treatment data and compliance. Two staff members from the project team will review treatment compliance and usage statistics weekly.

## 4.4 Schedule and Ethical Consideration

The study on asthenopia in individuals with presbyopia aims to begin enrollment immediately after receiving ethics approval from the Hong Kong Polytechnic University's Ethics Committee. This approach guarantees uniform ethical standards and participant protection measures.

## 4.5 Study Methodology

### 4.5.1 Study Procedure

- Eligibility: The research team will conduct visual acuity tests, slit lamp examinations, accommodation function examinations, and fundus examinations on the subjects to verify their eligibility for the study. Then informed consent will be obtained from the eligible subjects. Sociodemographic information and general health history will also be recorded.
- Asthenopia measurement: The asthenopia questionnaire scores will be derived from the Computer Vision Syndrome Questionnaire (CVS-Q), a widely recognized tool for assessing subjective symptoms of asthenopia. Change in CFF and other objective asthenopia indicators (e.g., eye movement, pupil parameters, and blink patterns) will be measured by a non-invasive eye tracker at 2 weeks and 1 month compared to baseline.
- Accommodation function: AA, AF, BCC, NRA, PRA, and AC/A ratio will be evaluated by optometrists at baseline, 2 weeks, and 1 month.
- Near visual ability: The NAVQ scores will be derived from the Near Activity Visual Questionnaire, a widely recognized tool for assessing near-vision functioning in presbyopia. HNVA was assessed at 40 cm using a near LogMAR chart under the participant’s habitual near correction. ADD was determined as the minimum additional lens power required to achieve best near visual acuity under full distance correction.
- Visual acuity: Best-corrected distance visual acuity (BCDVA) and best-corrected near visual acuity (BCNVA) at 40 cm will be measured at baseline, 2 weeks, and 1 month.
- Cognitive tests: Cognition function will be measured by EEG and the MoCA at baseline, 2 weeks, and 1month.
- Emotional states: Self-reported emotional states will be assessed by the PANAS-X at baseline, 2 weeks, and 1 month.
- Fundus Examinations: At baseline, 2 weeks and 1 month, participants will undergo non-invasive fundus examinations using Optical Coherence Tomography (VG200) and Laser Speckle Flowgraphy (LSFG-NAVI).

The data collected for the baseline visit and the follow-up visits are showed in Table 1.

Table 1. Time and Events Schedule for Study Procedures

|  | **Visit 1 (baseline examinations)** | **Visit 2 (2 weeks)** | **Visit 3 (1month)** |
| --- | --- | --- | --- |
| Pre-screening criteria | √ |  |  |
| Informed consent | √ |  |  |
| Non-cycloplegic refraction | √ |  |  |
| Slit lamp examination | √ |  |  |
| BCDVA | √ | √ | √ |
| BCNVA | √ | √ | √ |
| HNVA | √ | √ | √ |
| EEG | √ | √ | √ |
| MoCA scale | √ | √ | √ |
| Accommodation amplitude | √ | √ | √ |
| Facility of accommodation | √ | √ | √ |
| Accommodative response (BCC) | √ | √ | √ |
| Relative accommodation (NRA and PRA) | √ | √ | √ |
| AC/A ratio | √ | √ | √ |
| CVS-Q questionnaire | √ | √ | √ |
| NAVQ questionnaire | √ | √ | √ |
| CFF test | √ | √ | √ |
| Eye tracker test | √ | √ | √ |
| PANAS-X questionnaire | √ | √ | √ |
| Optical Coherence Tomography | √ | √ | √ |
| Laser Speckle Flowgraphy | √ | √ | √ |

### 4.5.2 Data Anonymity and Security

The information supplied by participants constitutes the research data. Any research data that can be used to identify participants is classified as personal data. Personal data does not include data where the identity has been removed (anonymous data). We will minimize our use of personal data in the study as much as possible. The researchers and their team, supervisors, collaborators will have access to personal and research data solely for study purposes. Responsible members of The Hong Kong Polytechnic University may be given access for monitoring and/or audit of the research.

All information related to the participants will remain confidential and be securely encrypted and stored with de-identification to ensure privacy. Confidentiality is mandated by agreements for all parties involved. The information collected will be kept until 5 years after project completion. The Hong Kong Polytechnic University takes reasonable precautions to prevent the loss, misappropriation, unauthorized access or destruction of the information that participants provide.

# **5. Participants Safety and Withdrawal**

## 5.1 Risk Management and Safety

The primary risk of the intervention is related to the low-level red-light therapy. The RLRL therapy device (Eyerising International Pty Ltd, Melbourne, Australia) is a semi-conductor laser product, with a power of 0.29 mW for a 4-mm pupil. It holds CE certification in the European Union, MHS registration in Malaysia, TGA approval in Australia, MHRA approval in the UK, medical device registration in Turkey, Medsafe registration in New Zealand, and a certificate from the National Medical Products Administration (NMPA) in China for myopia treatment. The manufacturer is certified under the ISO 13485 quality management system for medical device production. The device is widely available and commonly used for amblyopia treatment. Studies on the efficacy of RLRL for juvenile myopia control have been conducted in China, with no reported ocular complications or adverse effects in clinical trials.

Common side effects of the light therapy device include flash blindness, short-term glare, and afterimages, which typically resolve after a few minutes of eye closure. The examinations in this study are standard clinical procedures widely used in practice and research, posing minimal to no risk.

Adherence to task guidelines will be closely supervised, with active monitoring for adverse events. Any incidents will be promptly documented and addressed. Safety data, including discomfort, side effects, or health concerns, will be systematically collected and reviewed. Participants will be fully informed of potential risks and safety protocols.

## 5.2 Handling of Withdrawals

Study participation will be terminated by an investigator for the following reasons:

1. Any severe adverse event, such as a sudden loss of vision greater than two lines or the development of a central visual field scotoma.
2. The participant meets an exclusion criterion (either newly developed or previously unidentified) that precludes further involvement.
3. Any other reason deemed appropriate by the investigator.
4. New safety information necessitates halting the study.

Participants may withdraw from the study at any time upon request. Those who withdraw will be asked about their reasons and whether permission is granted to use data collected prior to withdrawal. If permission is denied, all associated data will be deleted from the system.

## 5.3 Replacement

Drop out will not be replaced.

## 5.4 Protocol Waivers or Deviations Justification

Protocol deviation is not allowed.

# **6. Statistical Methods**

## 6.1 Sample Size Estimation & Justification

The primary outcome was the change in Computer Vision Syndrome Questionnaire (CVS-Q) score at the 31-day follow-up visit. Due to the absence of prior randomized trials on RLRL therapy for presbyopia, the sample size estimation was informed by feasibility-based assumptions. We assumed a standardized mean difference of Cohen’s d = 0.80. Using a two-sided alpha of 0.05 and 80% power, calculations performed in G*Power 3.1 indicated a requirement of 26 participants per group (total n = 52). Allowing for a 15% loss to follow-up, the final target enrollment was 31 participants per group, totaling 62.

## 6.2 Power Calculations

Please refer to 6.1 Sample Size Estimation & Justification.

## 6.3 Statistical Methods to Be Undertaken

The normality of continuous variables will be assessed using the Kolmogorov–Smirnov test. Categorical variables will be summarized as counts and percentages (n [%]), and continuous variables as means ± standard deviations (SD). For comparisons of demographic or baseline characteristics between groups, independent samples t-tests will be used where assumptions are met. If data are not normally distributed or are ordinal, non-parametric tests such as the Mann–Whitney U test (for independent samples) and the Wilcoxon signed-rank test (for paired samples) will be applied. Outcomes were analyzed using both intention-to-treat (including all randomized participants) and per-protocol (including participants who completed both follow-up visits) approaches. To assess treatment effects over time, general linear mixed-effects models will be employed to account for repeated measures across multiple follow-up time points.

All statistical tests will be two-tailed with a significance level of α=0.05. Effect sizes, accompanied by 95% confidence intervals, will be reported to quantify the practical significance of the findings.

# **7. Storage of Blood and Tissue Samples**

Not applicable.

# **8. Data Security & Handling**

## 8.1 Where Records Will Be Kept & How Long Will They Be Stored

All data will be stored and backed up on secure servers at The Hong Kong Polytechnic University for 5 years. Please refer to 4.5.2 Data anonymity and security for more details.

## 8.2 Confidentiality and Security

According to the relevant agreement, all parties involved must keep study data confidential throughout the study process. All data related to the test cannot be accessed without authorization. The private information of research subjects will be protected in the report and the publication of any clinical research data.

## 8.3 Ancillary Data

All results captured will be transferred and stored on an encrypted, secured database. All data will be de-identified. Personal information and data will be kept secure at all times. Participant’s information will only be used for the purpose of this research project and it will only be disclosed with participant’ permission, except as required by law.

# **9. References**

1. Wolffsohn JS, Lingham G, Downie LE, et al. TFOS Lifestyle: Impact of the digital environment on the ocular surface. *Ocul Surf*. Apr 2023;28:213-252. doi:10.1016/j.jtos.2023.04.004

2. Hayes JR, Sheedy JE, Stelmack JA, Heaney CA. Computer use, symptoms, and quality of life. *Optom Vis Sci*. Aug 2007;84(8):738-44. doi:10.1097/OPX.0b013e31812f7546

3. Association OGoCO. Chinese expert consensus on the diagnosis and treatment of asthenopia (2024). *[Zhonghua yan ke za zhi] Chinese journal of ophthalmology*. 2024;60(4):322-329.

4. Lin N, Zhu Y, Wu X, Yang M, Lu F, Deng R. Prevalence and determinants of asthenopia among ophthalmologists in China: a national cross-sectional survey. *Front Public Health*. 2023;11:1290811. doi:10.3389/fpubh.2023.1290811

5. Galindo-Romero C, Rodríguez-Zamora CL, García-Ayuso D, Di Pierdomenico J, Valiente-Soriano FJ. Computer vision syndrome-related symptoms in presbyopic computer workers. *Int Ophthalmol*. Sep 2023;43(9):3237-3245. doi:10.1007/s10792-023-02724-z

6. Lin J, Ding X, Hong C, et al. Several biological benefits of the low color temperature light-emitting diodes based normal indoor lighting source. *Scientific reports*. 2019;9(1):7560.

7. Albarracin R, Valter K. 670 nm red light preconditioning supports Müller cell function: evidence from the white light‐induced damage model in the rat retina. *Photochemistry and Photobiology*. 2012;88(6):1418-1427.

8. Zhu L, Yang J, Yang X, Qin F, Wu D, Huang G. [Efficacy on visual display terminal syndrome treated with jingjin therapy of Zhuang medicine]. *Zhongguo Zhen Jiu*. Feb 12 2017;37(2):181-184. doi:10.13703/j.0255-2930.2017.02.020

9. Xiong Y, Wan XH, Li J, Li SM, Zhen Y. An experimental study of the protective effects of Chinese medicine compound eye-patch on asthenopia. *Chin J Integr Med*. Feb 2013;19(2):127-31. doi:10.1007/s11655-012-1217-5

10. Jiang Y, Zhu Z, Tan X, et al. Effect of Repeated Low-Level Red-Light Therapy for Myopia Control in Children: A Multicenter Randomized Controlled Trial. *Ophthalmology*. May 2022;129(5):509-519. doi:10.1016/j.ophtha.2021.11.023

11. Chen H, Wang W, Liao Y, et al. Low-intensity red-light therapy in slowing myopic progression and the rebound effect after its cessation in Chinese children: a randomized controlled trial. *Graefes Arch Clin Exp Ophthalmol*. Feb 2023;261(2):575-584. doi:10.1007/s00417-022-05794-4

12. Zhou L, Xing C, Qiang W, Hua C, Tong L. Low-intensity, long-wavelength red light slows the progression of myopia in children: an Eastern China-based cohort. *Ophthalmic Physiol Opt*. Mar 2022;42(2):335-344. doi:10.1111/opo.12939

13. Liu Z, Sun Z, Du B, et al. The Effects of Repeated Low-Level Red-Light Therapy on the Structure and Vasculature of the Choroid and Retina in Children with Premyopia. *Ophthalmol Ther*. Mar 2024;13(3):739-759. doi:10.1007/s40123-023-00875-x

14. Hamblin MR. Photobiomodulation for traumatic brain injury and stroke. *J Neurosci Res*. Apr 2018;96(4):731-743. doi:10.1002/jnr.24190

15. Hamblin MR. Shining light on the head: Photobiomodulation for brain disorders. *BBA Clinical*. 2016/12/01/ 2016;6:113-124. doi:<https://doi.org/10.1016/j.bbacli.2016.09.002>

16. Seguí Mdel M, Cabrero-García J, Crespo A, Verdú J, Ronda E. A reliable and valid questionnaire was developed to measure computer vision syndrome at the workplace. *J Clin Epidemiol*. Jun 2015;68(6):662-73. doi:10.1016/j.jclinepi.2015.01.015

17. Sharma G, Chiva-Razavi S, Viriato D, et al. Patient-reported outcome measures in presbyopia: a literature review. *BMJ Open Ophthalmol*. 2020;5(1):e000453. doi:10.1136/bmjophth-2020-000453

18. Hobson J. The montreal cognitive assessment (MoCA). *Occupational Medicine*. 2015;65(9):764-765.

19. Barrett DW, Gonzalez-Lima F. Transcranial infrared laser stimulation produces beneficial cognitive and emotional effects in humans. *Neuroscience*. 2013;230:13-23.
