# Supplementary material for: Repeated low-level red-light therapy for improving asthenopic symptoms and accommodation in presbyopia
Source: Ann Med. 2026 Apr 28;58(1):2663105. doi: 10.1080/07853890.2026.2663105 (PMC13126939; doi:10.1080/07853890.2026.2663105)
Supplement: Supplement 2_Supplementary Online Content.docx [file IANN_A_2663105_SM8667.docx]

**Supplement 2**

**Supplementary Online Content**

Table S1. Subgroup analysis of adjusted mean change in accommodative amplitude (AA) by baseline AA level.

Table S2. Generalized linear mixed-effects model (GLMM) results for changes in amplitude of accommodation (AA), accommodative facility (AF), and NAVQ score, adjusted for the baseline value of the outcome, age, and gender.

Table S3. Generalized linear mixed-effects model (GLMM) results for changes in amplitude of accommodation (AA), accommodative facility (AF), and NAVQ score, adjusted for the baseline value of the outcome, age, gender, and baseline spherical equivalent (SER).

Table S4. Unadjusted Mean Value of Outcomes and Within-Group Comparisons Over Time.

Table S5. Cumulative adjusted mean changes in NAVQ score from baseline to 15 and 31 days in RLRL group and Sham group.

Table S6. Unadjusted mean values and mean changes in near visual ability measures in RLRL and Sham groups.

Table S7. Intervention compliance in RLRL and Sham groups.

Figure S1. Adjusted mean difference (RLRL − Sham) in change from baseline in Computer Vision Syndrome Questionnaire (CVS-Q) score at follow-up visits.

Appendix S1. Prespecified secondary outcomes: collection and reporting.

This supplementary material has been provided by the authors to give readers additional information about their work.

Table S1. Subgroup analysis of adjusted mean change in accommodative amplitude (AA) by baseline AA level. Values are adjusted means (95% CI), mean differences (95% CI), and P values from generalized linear mixed models (GLMM). CI = confidence interval.

| Outcomes | Visit | Adjusted mean  (95% CI) | | Mean Difference  (95% CI) | P Value |
| --- | --- | --- | --- | --- | --- |
|  |  | RLRL Arm | Sham Arm |  |  |
| **Secondary Outcome** | | | | | |
| **Change of AA (OD), D** | | | | | |
| >2D | 15 days | 0.38 (-0.39, 1.14) | -0.79 (-1.53, -0.05) | 1.17 (0.10, 2.23) | 0.032 |
| >2D | 31 days | 0.70 (-0.07, 1.46) | -0.70 (-1.44, 0.05) | 1.39 (0.32, 2.46) | 0.011 |
| ≤2D | 15 days | 0.86 (0.29, 1.43) | 1.16 (0.56, 1.76) | -0.30 (-1.13, 0.52) | 0.472 |
| ≤2D | 31 days | 1.17 (0.61, 1.74) | 1.32 (0.72, 1.92) | -0.15 (-0.97, 0.68) | 0.727 |
| **Change of AA (OS), D** | | | | | |
| >2D | 15 days | 0.63 (-0.08, 1.35) | -0.93 (-1.53, -0.32) | 1.56 (0.62, 2.49) | 0.001 |
| >2D | 31 days | 0.85 (0.13, 1.57) | -0.88 (-1.48, -0.28) | 1.73 (0.79, 2.67) | <0.001 |
| ≤2D | 15 days | 0.72 (0.13, 1.32) | 1.40 (0.55, 2.26) | -0.68 (-1.72, 0.36) | 0.202 |
| ≤2D | 31 days | 1.51 (0.91, 2.11) | 1.98 (1.12, 2.84) | -0.47 (-1.52, 0.58) | 0.380 |
| **Change of AA (OU), D** | | | | | |
| >2D | 15 days | 0.34 (-0.39, 1.06) | -0.71 (-1.41, -0.01) | 1.05 (0.04, 2.06) | 0.042 |
| >2D | 31 days | 0.97 (0.24, 1.69) | -0.36 (-1.06, 0.33) | 1.33 (0.32, 2.34) | 0.010 |
| ≤2D | 15 days | 2.05 (1.02, 3.09) | 2.42 (1.20, 3.64) | -0.37 (-1.97, 1.24) | 0.652 |
| ≤2D | 31 days | 3.03 (2.00, 4.07) | 2.23 (0.99, 3.47) | 0.80 (-0.81, 2.42) | 0.329 |

Table S2. Generalized linear mixed-effects model (GLMM) results for changes in amplitude of accommodation (AA), accommodative facility (AF), and NAVQ score, adjusted for the baseline value of the outcome, age, and gender. M/F = male/female; D = diopter.

| **Factors** | **Coefficient (95% CI)** | **P Value** |
| --- | --- | --- |
| **Primary outcome** |  |  |
| **Change of CVS-Q score** |  |  |
| Age, year | 0.03 (-0.06, 0.12) | 0.526 |
| Gender (F/M) | -0.35 (-1.41, 0.70) | 0.515 |
| Baseline CVS-Q score | -0.24 (-0.36, -0.12) | <0.001 |
| Follow-up 1 vs Baseline (Sham) | -0.50 (-1.68, 0.68) | 0.409 |
| Follow-up 2 vs Baseline (Sham) | -0.79 (-1.97, 0.39) | 0.190 |
| RLRL vs Sham at Baseline | 0.09 (-1.27, 1.45) | 0.893 |
| RLRL vs Sham at Follow-up 1 | -1.41 (-3.08, 0.26) | 0.097 |
| RLRL vs Sham at Follow-up 2 | -1.87 (-3.54, -0.20) | 0.028 |
| **Secondary outcome** |  |  |
| **Change of AA (OD), D** |  |  |
| Age, year | -0.036 (-0.073, 0.001) | 0.058 |
| Gender (F/M) | 0.342 (-0.084, 0.768) | 0.116 |
| Baseline AA, D | -0.313 (-0.432, -0.194) | <0.001 |
| Follow-up 1 vs Baseline (Sham) | -0.067 (-0.615, 0.480) | 0.809 |
| Follow-up 2 vs Baseline (Sham) | 0.057 (-0.490, 0.604) | 0.838 |
| RLRL vs Sham at Baseline | -0.147 (-0.739, 0.445) | 0.626 |
| RLRL vs Sham at Follow-up 1 | 0.650 (-0.124, 1.424) | 0.100 |
| RLRL vs Sham at Follow-up 2 | 0.842 (0.068, 1.616) | 0.033 |
| **Change of AA (OS), D** |  |  |
| Age, year | -0.027 (-0.064, 0.009) | 0.142 |
| Gender (F/M) | 0.485 (0.063, 0.907) | 0.024 |
| Baseline AA, D | -0.378 (-0.494, -0.263) | <0.001 |
| Follow-up 1 vs Baseline (Sham) | -0.425 (-0.967, 0.116) | 0.124 |
| Follow-up 2 vs Baseline (Sham) | -0.260 (-0.801, 0.282) | 0.347 |
| RLRL vs Sham at Baseline | -0.314 (-0.905, 0.277) | 0.297 |
| RLRL vs Sham at Follow-up 1 | 1.108 (0.342, 1.874) | 0.005 |
| RLRL vs Sham at Follow-up 2 | 1.409 (0.643, 2.175) | <0.001 |
| **Change of AA (OU), D** |  |  |
| Age, year | -0.032 (-0.075, 0.012) | 0.151 |
| Gender (F/M) | 0.258 (-0.249, 0.766) | 0.318 |
| Baseline AA, D | -0.366 (-0.473, -0.259) | <0.001 |
| Follow-up 1 vs Baseline (Sham) | -0.239 (-0.907, 0.429) | 0.483 |
| Follow-up 2 vs Baseline (Sham) | 0.008 (-0.660, 0.676) | 0.981 |
| RLRL vs Sham at Baseline | -0.311 (-1.026, 0.404) | 0.394 |
| RLRL vs Sham at Follow-up 1 | 0.946 (0.002, 1.890) | 0.050 |
| RLRL vs Sham at Follow-up 2 | 1.405 (0.461, 2.350) | 0.004 |
| **Change of AF (OU), cpm** |  |  |
| Age, year | -0.048 (-0.107, 0.012) | 0.116 |
| Gender (F/M) | 0.243 (-0.426, 0.912) | 0.476 |
| Baseline AF, D | -0.537 (-0.639, -0.435) | <0.001 |
| Follow-up 1 vs Baseline (Sham) | 0.266 (-0.717, 1.250) | 0.596 |
| Follow-up 2 vs Baseline (Sham) | 1.269 (0.286, 2.253) | 0.011 |
| RLRL vs Sham at Baseline | -0.581 (-1.587, 0.425) | 0.258 |
| RLRL vs Sham at Follow-up 1 | 1.586 (0.195, 2.976) | 0.025 |
| RLRL vs Sham at Follow-up 2 | 0.989 (-0.402, 2.380) | 0.163 |
| **Change of NAVQ score** |  |  |
| Age, year | 0.24 (-0.17, 0.65) | 0.249 |
| Gender (F/M) | -0.52 (-5.21, 4.17) | 0.829 |
| Baseline NAVQ score | -0.28 (-0.41, -0.16) | <0.001 |
| Follow-up 1 vs Baseline (Sham) | -2.38 (-7.55, 2.78) | 0.366 |
| Follow-up 2 vs Baseline (Sham) | -3.03 (-8.20, 2.14) | 0.250 |
| RLRL vs Sham at Baseline | -0.81 (-6.83, 5.20) | 0.791 |
| RLRL vs Sham at Follow-up 1 | -5.88 (-13.18, 1.43) | 0.115 |
| RLRL vs Sham at Follow-up 2 | -7.26 (-14.57, 0.05) | 0.051 |

Table S3. Generalized linear mixed-effects model (GLMM) results for changes in amplitude of accommodation (AA), accommodative facility (AF), and NAVQ score, adjusted for the baseline value of the outcome, age, gender, and baseline spherical equivalent (SER). For OD/OS, SER is eye-specific; for OU, SER is the mean of the two eyes. M/F = male/female; D = diopter.

| **Factors** | **Coefficient (95% CI)** | **P Value** |
| --- | --- | --- |
| **Primary outcome** |  |  |
| **Change of CVS-Q score** |  |  |
| Age, year | 0.02 (-0.08, 0.12) | 0.714 |
| Gender (F/M) | -0.17 (-1.34, 1.00) | 0.773 |
| SER, D | 0.03 (-0.19, 0.25) | 0.819 |
| Baseline AA, D | 0.04 (-0.21, 0.28) | 0.770 |
| Follow-up 1 vs Baseline (Sham) | -0.46 (-1.64, 0.72) | 0.441 |
| Follow-up 2 vs Baseline (Sham) | -0.75 (-1.93, 0.43) | 0.213 |
| RLRL vs Sham at Baseline | 0.05 (-1.40, 1.50) | 0.948 |
| RLRL vs Sham at Follow-up 1 | -1.44 (-3.11, 0.23) | 0.091 |
| RLRL vs Sham at Follow-up 2 | -1.90 (-3.57, -0.24) | 0.025 |
| **Secondary outcome** |  |  |
| **Change of AA (OD), D** |  |  |
| Age, year | -0.034 (-0.072, 0.004) | 0.080 |
| Gender (F/M) | 0.329 (-0.101, 0.759) | 0.134 |
| SER, D | -0.016 (-0.094, 0.061) | 0.680 |
| Baseline AA, D | -0.317 (-0.437, -0.197) | <0.001 |
| Follow-up 1 vs Baseline (Sham) | -0.068 (-0.616, 0.479) | 0.807 |
| Follow-up 2 vs Baseline (Sham) | 0.056 (-0.492, 0.603) | 0.842 |
| RLRL vs Sham at Baseline | -0.158 (-0.751, 0.436) | 0.603 |
| RLRL vs Sham at Follow-up 1 | 0.650 (-0.124, 1.425) | 0.100 |
| RLRL vs Sham at Follow-up 2 | 0.843 (0.069, 1.618) | 0.033 |
| **Change of AA (OS), D** |  |  |
| Age, year | -0.026 (-0.064, 0.011) | 0.168 |
| Gender (F/M) | 0.481 (0.058, 0.904) | 0.026 |
| SER, D | -0.011 (-0.091, 0.070) | 0.796 |
| Baseline AA, D | -0.380 (-0.497, -0.264) | <0.001 |
| Follow-up 1 vs Baseline (Sham) | -0.426 (-0.967, 0.116) | 0.123 |
| Follow-up 2 vs Baseline (Sham) | -0.260 (-0.802, 0.281) | 0.346 |
| RLRL vs Sham at Baseline | -0.320 (-0.913, 0.273) | 0.290 |
| RLRL vs Sham at Follow-up 1 | 1.109 (0.343, 1.874) | 0.005 |
| RLRL vs Sham at Follow-up 2 | 1.410 (0.644, 2.176) | <0.001 |
| **Change of AA (OU), D** |  |  |
| Age, year | -0.024 (-0.068, 0.020) | 0.289 |
| Gender (F/M) | 0.220 (-0.281, 0.721) | 0.389 |
| SER, D | -0.072 (-0.165, 0.021) | 0.127 |
| Baseline AA, D | -0.369 (-0.475, -0.264) | <0.001 |
| Follow-up 1 vs Baseline (Sham) | -0.243 (-0.911, 0.426) | 0.477 |
| Follow-up 2 vs Baseline (Sham) | 0.004 (-0.665, 0.672) | 0.992 |
| RLRL vs Sham at Baseline | -0.348 (-1.060, 0.363) | 0.337 |
| RLRL vs Sham at Follow-up 1 | 0.947 (0.003, 1.892) | 0.049 |
| RLRL vs Sham at Follow-up 2 | 1.408 (0.463, 2.352) | 0.003 |
| **Change of AF (OU), cpm** |  |  |
| Age, year | 0.061 (-0.032, 0.155) | 0.200 |
| Gender (F/M) | -0.319 (-1.369, 0.731) | 0.552 |
| SER, D | -0.163 (-0.360, 0.035) | 0.106 |
| Baseline AF, cycles/30 s | 0.032 (-0.188, 0.253) | 0.775 |
| Follow-up 1 vs Baseline (Sham) | 0.284 (-0.704, 1.272) | 0.573 |
| Follow-up 2 vs Baseline (Sham) | 1.273 (0.285, 2.261) | 0.012 |
| RLRL vs Sham at Baseline | -0.040 (-1.307, 1.227) | 0.951 |
| RLRL vs Sham at Follow-up 1 | 1.559 (0.163, 2.956) | 0.029 |
| RLRL vs Sham at Follow-up 2 | 0.977 (-0.420, 2.374) | 0.171 |
| **Change of NAVQ score** |  |  |
| Age, year | 0.155 (-0.323, 0.633) | 0.525 |
| Gender (F/M) | -0.616 (-5.980, 4.747) | 0.822 |
| SER, D | 0.327 (-0.680, 1.334) | 0.525 |
| Baseline NAVQ | 0.242 (-0.884, 1.368) | 0.674 |
| Follow-up 1 vs Baseline (Sham) | -2.396 (-7.565, 2.774) | 0.364 |
| Follow-up 2 vs Baseline (Sham) | -3.047 (-8.218, 2.124) | 0.248 |
| RLRL vs Sham at Baseline | 0.402 (-6.128, 6.932) | 0.904 |
| RLRL vs Sham at Follow-up 1 | -6.046 (-13.355, 1.263) | 0.105 |
| RLRL vs Sham at Follow-up 2 | -7.426 (-14.737, -0.116) | 0.046 |

Table S4. Unadjusted Mean Value of Outcomes and Within-Group Comparisons Over Time.

| Outcome | Group | Baseline  mean ± SD | 15 Days  mean ± SD | 31 Days  mean ± SD | P Value  (Baseline vs 15 Days) | P Value  (Baseline vs 31 Days) |
| --- | --- | --- | --- | --- | --- | --- |
| AA (OD) | RLRL | 2.24 (1.43) | 2.79 (2.19) | 3.10 (1.74) | 0.050 | <0.001 |
|  | Sham | 2.69 (2.10) | 2.67 (1.62) | 2.78 (1.88) | 0.993 | 0.629 |
| AA (OS) | RLRL | 2.21 (1.31) | 2.86 (2.01) | 3.31 (1.81) | 0.018 | <0.001 |
|  | Sham | 3.03 (2.14) | 2.66 (1.41) | 2.80 (1.63) | 0.256 | 0.330 |
| AA (OU) | RLRL | 3.05 (1.96) | 3.75 (2.09) | 4.43 (2.09) | 0.031 | <0.001 |
|  | Sham | 3.88 (2.60) | 3.71 (1.92) | 3.94 (2.28) | 0.644 | 0.768 |
| AF (OU) | RLRL | 6.68 (3.46) | 8.47 (2.40) | 8.86 (2.45) | 0.013 | 0.001 |
|  | Sham | 7.74 (2.86) | 8.02 (2.12) | 8.98 (2.24) | 0.483 | 0.029 |
| CVS-Q | RLRL | 9.15 (4.62) | 7.30 (4.26) | 6.58 (3.78) | 0.012 | <0.001 |
|  | Sham | 8.79 (3.50) | 8.33 (4.32) | 8.06 (4.96) | 0.476 | 0.259 |
| NAVQ | RLRL | 46.67 (17.99) | 38.47 (16.79) | 36.50 (14.70) | 0.036 | 0.002 |
|  | Sham | 49.75 (17.70) | 47.40 (20.46) | 46.78 (19.60) | 0.386 | 0.261 |
| NRA | RLRL | 1.66 (0.77) | 1.89 (0.70) | 2.07 (0.56) | 0.014 | 0.002 |
|  | Sham | 1.81 (0.84) | 1.88 (0.62) | 2.20 (0.67) | 0.369 | 0.047 |
| PRA | RLRL | 0.66 (0.51) | 0.71 (0.57) | 0.67 (0.45) | 0.614 | 0.885 |
|  | Sham | 0.69 (0.53) | 0.49 (0.51) | 0.68 (0.45) | 0.073 | 0.937 |
| BCC | RLRL | 1.08 (0.82) | 1.52 (0.75) | 1.67 (0.75) | 0.002 | <0.001 |
|  | Sham | 1.25 (0.78) | 1.82 (0.58) | 1.87 (0.58) | <0.001 | <0.001 |
| AC/A | RLRL | 3.21 (2.84) | 2.69 (2.21) | 1.84 (1.80) | 0.165 | 0.010 |
|  | Sham | 2.15 (2.95) | 1.85 (2.02) | 1.70 (2.16) | 0.934 | 0.740 |

Table S5. Cumulative adjusted mean changes in NAVQ score from baseline to 15 and 31 days in RLRL group and Sham group. Values are from generalized linear mixed models (GLMMs) adjusted for baseline score, age, and sex. Mean differences represent between-group comparisons at each visit. NAVQ = Near Activity Visual Questionnaire; CI = confidence interval.

| Outcome | Visit/Group | Cumulative adjusted mean change of  outcomes (95% CI) | | Mean Difference  (95% CI) | P Value |
| --- | --- | --- | --- | --- | --- |
|  |  | RLRL Arm | Sham Arm |  |  |
| NAVQ | 15 Days | -8.77 (-13.08, -4.46) | -2.09 (-6.40, 2.22) | -6.69 (-12.79, -0.59) | 0.032 |
|  | 31 Days | -10.80 (-15.11, -6.50) | -2.73 (-7.04, 1.58) | -8.07 (-14.17, -1.97) | 0.009 |

Table S6. Unadjusted mean values and mean changes in near visual ability measures in RLRL and Sham groups. P values are from Wilcoxon rank-sum tests. HNVA = habitual near visual acuity; ADD = near addition; NAVQ = Near Activity Visual Questionnaire; D = diopters; SD = standard deviation.

| Outcome | Mean (SD) | | P Value |
| --- | --- | --- | --- |
|  | RLRL Arm | Sham Arm |  |
| **HNVA** |  |  |  |
| Baseline | 0.12 (0.12) | 0.13 (0.17) | 0.571 |
| At 15 days | 0.08 (0.10) | 0.10 (0.15) | 0.791 |
| At 31 days | 0.06 (0.10) | 0.09 (0.14) | 0.570 |
| Change at 15 days | -0.04 (0.09) | -0.03 (0.08) | 0.591 |
| Change at 31 days | -0.06 (0.09) | -0.04 (0.10) | 0.124 |
| **ADD, D** |  |  |  |
| Baseline | 1.70 (0.53) | 1.72 (0.56) | 0.866 |
| At 15 days | 1.66 (0.52) | 1.76 (0.56) | 0.460 |
| At 31 days | 1.69 (0.53) | 1.77 (0.57) | 0.541 |
| Change at 15 days | -0.04 (0.19) | 0.04 (0.23) | 0.145 |
| Change at 31 days | -0.01 (0.23) | 0.05 (0.25) | 0.062 |
| **NAVQ** |  |  |  |
| Baseline | 46.67 (17.99) | 49.75 (17.70) | 0.485 |
| At 15 days | 38.47 (16.79) | 47.40 (20.46) | 0.057 |
| At 31 days | 36.50 (14.70) | 46.78 (19.60) | 0.062 |
| Change at 15 days | -8.20 (19.63) | -2.35 (15.36) | 0.206 |
| Change at 31 days | -10.17 (16.06) | -2.97 (14.89) | 0.086 |

Table S7. Intervention compliance in RLRL and Sham groups. P values are from Wilcoxon rank-sum tests. SD = standard deviation.

| Item | Sham Arm (n=32) | RLRL Arm (n=32) | P Value |
| --- | --- | --- | --- |
| Prescribed sessions | 62 | 62 |  |
| Completed sessions, mean ± SD | 60.4 ± 2.0 | 60.9 ± 2.0 | 0.149 |
| Completed sessions, range (min–max) | 54–62 | 55–62 |  |
| Compliance rate (%), mean ± SD | 97.5 ± 3.2 | 98.2 ± 3.2 | 0.149 |
| Compliance rate (%), range (min–max) | 87.1–100.0 | 88.7–100.0 |  |
| Met compliance threshold (≥50/62, ≥80%), n/N (%) | 32/32 (100.0%) | 32/32 (100.0%) | Not applicable |


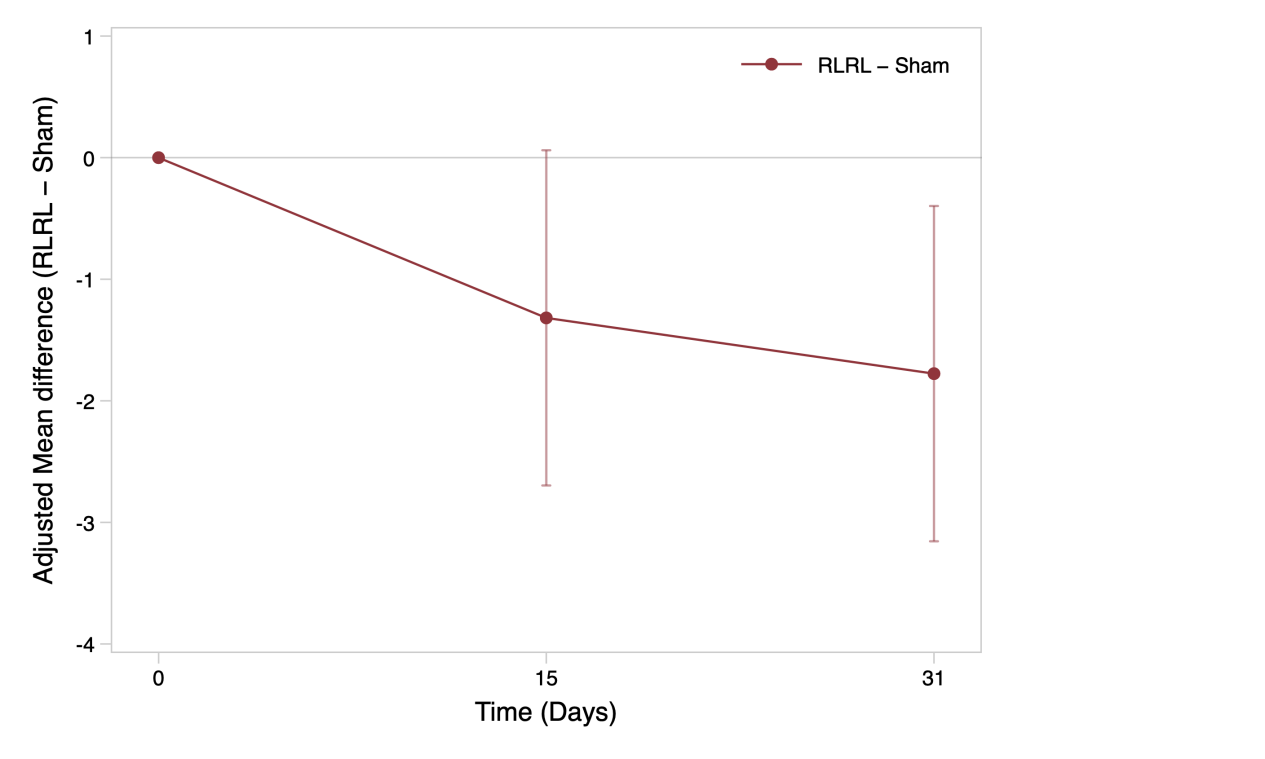


Figure S1. Adjusted mean difference (RLRL − Sham) in change from baseline in Computer Vision Syndrome Questionnaire (CVS-Q) score at follow-up visits. Error bars represent 95% confidence intervals (CIs). RLRL = repeated low-level red-light therapy.

Appendix S1. Other prespecified secondary outcomes: collection and reporting. CFF = critical flicker fusion (frequency); MoCA = Montreal Cognitive Assessment Scale; PANAS-X = Positive and Negative Affect Schedule; LSFG = laser speckle flowgraphy; OCT = optical coherence tomography;

OCTA = optical coherence tomography angiography; EEG = electroencephalography; CVS-Q = Computer Vision Syndrome Questionnaire.

To ensure transparent accounting of prespecified secondary outcomes, we summarize the collection status of objective secondary outcomes listed in the trial protocol.

1. **Collected prespecified outcomes (available)**: CFF, eye-tracking metrics (pupil, blink, and eye-movement parameters), MoCA, PANAS-X psychological metrics, LSFG perfusion imaging, and OCT/OCTA imaging were collected as prespecified.
2. **Not collected (and why)**: EEG was not collected due to staffing and budget constraints that prevented implementation of the EEG testing workflow.
3. **Collected but not included in the present report (and why)**: To maintain focus on the prespecified primary endpoint (CVS-Q) and key clinical secondary outcomes and to limit additional multiple comparisons and manuscript length, the following exploratory/ancillary outcomes are not reported in the present manuscript: CFF, eye-tracking metrics, MoCA, PANAS-X, and quantitative OCT/OCTA structural/vascular metrics (e.g., choroidal thickness and OCTA perfusion/vessel-density parameters). LSFG perfusion imaging was collected. However, a substantial proportion of scans did not meet predefined image-quality requirements, precluding reliable quantitative analysis. OCT/OCTA imaging in the present manuscript was used for structural safety review only.
